# Supplementary material for: WordCommentsAnalyzer: A windows software tool for qualitative research
Source: F1000Res. 2018 Sep 4;7:536. Originally published 2018 May 3. [Version 2] doi: 10.12688/f1000research.14819.2 (PMC6137411; doi:10.12688/f1000research.14819.2)
Supplement: Supplementary file 3 [file f1000research-7-17614-s0002.tgz › b2a79496-4ada-47c4-9682-a99ce867836d.zip!/Eight Iranian Health Journals/Journal of Research in Rehabilitation Sciences _12.docx]

© Journal of Research in Rehabilitation Sciences . This work is licensed under CC BY-NC 4.0 (https://creativecommons.org/licenses/by-nc/4.0/).

Volume & Issue: دوره 11، شماره 5: 1394

Title: تأثیر هشت هفته تمرینات ریباندتراپی بر روی تعادل، انعطاف‌پذیری و قدرت عضلات زانوی کودکان مبتلا به فلج مغزی اسپاستیک

http://jrrs.mui.ac.ir/index.php/jrrs/article/view/2138

مقدمه: فلج مغزی شایع‌ترین علت ناتوانی‌های حرکتی در دوران کودکی است. مطالعه حاضر با هدف تعيين تأثير 8 هفته تمرینات درماني ریباندتراپی بر تعادل، انعطاف‌پذیری و قدرت عضلات زانوی کودکان مبتلا به فلج مغزی انجام گرفت. مواد و روش‌ها: در اين پژوهش نيمه تجربي، 20 کودک 6 تا 12 ساله مبتلا به فلج مغزی اسپاستیک مشغول به تحصیل در مدارس جسمی- حرکتی یا در مراکز آموزشی کودکان استثنایی شهر اصفهان، به روش در دسترس هدفمند انتخاب شدند. بیماران به صورت تصادفي جفت شده در دو گروه شاهد (10 نفر) و تجربی (10 نفر) قرار گرفتند. پس از يك جلسه توجيهي، از والدین همه آزمودني‌ها رضايت‌نامه كتبي اخذ گرديد. برای اندازه‌گیری قدرت دو گروه عضلانی راست کننده و خم کننده زانو، از دستگاه دینامومتر دیجیتال MIE medical research Ltd. (با دقت 1/0 کیلوگرم)، جهت اندازه‌گیری انعطاف‌پذیری عمومی از آزمون بشین و برس و برای اندازه‌گیری تعادل نیز از مقیاس تعادلی Berg استفاده گردید. بيماران به مدت 8 هفته و در هر هفته سه جلسه، تمرينات منتخب را تحت نظر متخصص انجام دادند. جهت تجزیه و تحليل داده‌ها، روش آماري تحليل واريانس براي داده‌هاي تکراري در سطح معنی‌داری 05/0 > P مورد استفاده قرار گرفت. یافته‌ها: تمام عوامل اندازه‌گيري شده در مطالعه، شامل تعادل، انعطاف‌پذیری و قدرت عضلات زانو در بيماران داراي تعامل معنی‌داری بود (05/0 > P)؛ بدین معنی كه از پيش‌آزمون تا پس‌آزمون، گروه ریباندتراپی پيشرفت بیشتری را نسبت به گروه شاهد نشان داد. نتیجه‌گیری: با توجه به نتایج به دست آمده مبنی بر اثربخش بودن برنامه تمرینی ریباندتراپی و همچنین، هزینه کم، بی‌خطر و قابل استفاده بودن آن در منزل، این تمرینات می‌تواند مورد استفاده طیف وسیعی از مبتلایان به فلج مغزی قرار گیرد.

Title: بررسی تأثیر تمرینات درکی- حرکتی بر زبردستی کودکان مبتلا به سندرم داون (طرح تک‌آزمودنی

http://jrrs.mui.ac.ir/index.php/jrrs/article/view/2225

مقدمه: سندرم داون به عنوان یک اختلال ژنتیکی، از عوامل عمده عقب ماندگی ذهنی است که منجر به تأخیر در مهارت‌های حرکتی از قبیل حرکات ظریف، حرکات درشت، قدرت و کنترل بینایی- حرکتی می‌گردد. زبردستی یک مهارت عملکردی دست می‌باشد و نیازمند هماهنگی حرکات درشت و ظریف است که تحت تأثیر مشکلات حرکتی در این کودکان قرار می‌گیرد. نظر به اهمیت توسعه حرکات درشت، ظریف و هماهنگی بین آن‌ها جهت اجرای فعالیت‌هایی مانند زبردستی، هدف از انجام مطالعه حاضر، بررسی تأثیر تمرینات درکی- حرکتی بر زبردستی کودکان مبتلا به سندرم داون بود. مواد و روش‌ها: این مطالعه به صورت کارآزمایی بالینی از نوع طرح تک‌آزمودنی با استفاده از نمونه در دسترس انجام گرفت. شرکت ‌کننده‌ها در فاز پایه به صورت دو بار در هفته با آزمون Purdue Pegboard مورد ارزیابی قرار گرفتند. در فاز درمان به مدت 5/1 ماه، هر هفته 3 جلسه 60 دقیقه‌ای در تمرینات ادراكي- حرکتی شرکت کردند. جهت بررسی الگوی تغییرات در فاز پایه و درمان، از آماره C و آزمون Bayesian analyze استفاده گردید. یافته‌ها: طبق نتایج آزمون Bayesian analyze، تمرینات درکی- حرکتی بر زبردستی بر اساس میانگین نمرات مونتاژ (Mean montage score)، دارای تأثیر خیلی قوی در شرکت کنندگان 1 و 2، قوی تا خیلی قوی در شرکت کننده چهار، متوسط تا قوی در شرکت کننده پنج و تأثیر ضعیف در شرکت کننده سه بود. نتیجه‎گیری: تمرینات درکی- حرکتی می‌تواند مداخله مؤثری برای بهبود زبردستی کودکان مبتلا به سندرم داون باشد. از این‌رو، مطالعات با متدولوژی قوی‌تری لازم است.

Title: تأثیر تسهیل شرایط تمرینی دویدن بر خودکارامدی دویدن کودکان نابینا

http://jrrs.mui.ac.ir/index.php/jrrs/article/view/2301

مقدمه: در بسیاری از موارد شرایط جامعه به ‌گونه‌ای است که باعث کاهش فرصت‌های حرکتی، به ویژه فرصت تجربه موفق دویدن برای کودکان نابینا می‌شود. به دنبال این مسأله، خودکارامدی کودکان نابینا نسبت به دویدن که یک فعالیت بنیادی و مهم برای رشد حرکتي، جسماني و روانی در دوران کودکی است، کاهش می‌یابد. هدف از انجام پژوهش حاضر، بررسی تأثير تسهیل شرایط تمرینی دویدن بر خودکارامدی دویدن کودکان نابینا بود. مواد و روش‌ها: نمونه‌های تحقیق نیمه تجربی حاضر را كودكان نابينای یکی از مدارس ابتدایی نابینایان استان اصفهان تشکیل دادند که در دو گروه تجربی شامل 8 نفر (2 دختر و 6 پسر با میانگین سنی 6/1 ± 5/9 سال) و شاهد شامل 10 نفر (3 دختر و 7 پسر با میانگین سنی 6/1 ± 3/10 سال) قرار گرفتند. تسهیل شرایط دویدن با استفاده از روش «کمربند اتصالی برای گروهی دویدن نابینایان» انجام شد. برنامه تمرینی به صورت 3 جلسه 45 دقيقه‌ای در هفته در نظر گرفته شد و به مدت 6 هفته ادامه داشت. خودکارامدی دويدن با استفاده از پرسش‌نامه خودکارامدی دويدن نابينايان به شکل مصاحبه و به صورت پيش‌آزمون و پس‌آزمون اندازه‌گيري شد. جهت تحلیل داده‌ها، از روش آماری ANOVA برای داده‌های تکراری در سطح معنی‌داری 050/0 ≥ P استفاده گردید. یافته‌ها: یافته‌ها بیانگر تعامل معنی‌دار در خودکارامدی مسافت دويدن (020/0 = P و 35/6 = (16 و 1)F) و خودکارامدی مهارت دويدن (001/0 > P و 19/14 = (16 و 1)F) به نفع گروه تجربی بود. همچنین، اثر اصلي نوبت آزمون (تفاوت درون گروهی) در خودکارامدی مسافت دويدن (010/0 = P و 9/52 = (16 و 1)F) و خودکارامدی مهارت دويدن (001/0 > P و 56/20 = (16 و 1)F) به نفع گروه تجربي بود، اما اثر اصلي گروه (تفاوت بین گروهی) در هیچ کدام از خودکارامدی‌ها (مسافت دویدن و مهارت دویدن) معنی‌دار مشاهده نشد. نتیجه‎گیری: پيشرفت معنی‌دار گروه تجربي شاید به تقویت منابع خودکارامدی در نتیجه انجام تمرینات دویدن با شرایط تسهیل شده، مربوط می‌شود. اين نتایج نشان می‌دهد که در صورت تسهیل شرایط، تمرينات دويدن برای كودكان نابينا امكان‌پذير است و اثر مطلوبی در بهبود خودکارامدی دویدن آن‌ها دارد.

Title: تأثیر فوری کشش عضلانی غیر فعال بر میزان قند خون افراد مبتلا به دیابت نوع 2

http://jrrs.mui.ac.ir/index.php/jrrs/article/view/2555

مقدمه: شواهد جدید نشان دهنده تأثیر کشش عضلانی غیر فعال بر کاهش میزان قند خون است. هدف از انجام مطالعه حاضر، بررسی تأثیر فوری کشش غیر فعال بر روی میزان قند خون افراد مبتلا به دیابت بود. مواد و روش‌ها: در این مطالعه، 50 بیمار مبتلا به دیابت به صورت تصادفی به دو گروه مداخله (25 نفر) و شاهد (25 نفر) تقسیم شدند. افراد گروه مداخله 20 دقیقه کشش عضلانی غیر فعال دریافت کردند و افراد گروه شاهد هیچ کششی دریافت نکردند. میزان قند خون افراد قبل از مداخله و بلافاصله بعد از مداخله اندازه‌گیری گردید. یافته‌ها: سطح قند خون بلافاصله بعد از کشش در گروه مداخله نسبت به گروه شاهد کاهش معنی‌داری را نشان داد (001/0 > P). سطح قند خون در گروه شاهد قبل و بعد از مداخله تفاوت معنی‌داری داشت. نتیجه‌‌‌گیری: نتایج مطالعه حاضر حاکی از آن بود که کشش عضلانی غیر فعال اثر قابل توجهی در کاهش فوری سطح قند خون بیماران مبتلا به دیابت نوع 2 دارد.

Title: تأثیر مداخله زودهنگام مبتنی بر والدین بر مهارت‌های زبانی کودکان نوپای مبتلا به تأخیر زبانی: پژوهش مورد- منفرد

http://jrrs.mui.ac.ir/index.php/jrrs/article/view/2543

مقدمه: والدین کودکان مبتلا به آسیب‌های زبانی، نقش بسیار مهمی در بهبود رشد زبانی کودکشان ایفا می‌کنند. بنابراین، آموزش به آن‌ها در قالب مداخله زودهنگام، امری حیاتی است. از برنامه‌های مؤثر در این زمینه، مدل «آموزش فراگیر پیشرفته» می‌باشد که با اجرای استراتژی‌های آموزش زبان در بافت طبیعی تعاملات روزمره والد- کودک، باعث پیشرفت مهارت‌های ارتباطی کودکان مبتلا به آسیب‌های زبانی می‌شود. هدف از انجام مطالعه حاضر، بررسی تأثیر این برنامه بر مهارت‌های زبان بیانی کودکان نوپای ایرانی (30-24 ماه) مبتلا به تأخیر در صحبت کردن شهر اصفهان بود. مواد و روش‌ها: روش انجام مطالعه، طرح مورد منفرد از نوع خط پایه چندگانه با رفتارهای مختلف بود. 3 کودک نوپای مبتلا به تأخیر در شروع گفتار و مادرانشان، به صورت غیر تصادفی انتخاب شدند و به ترتیب طی 17، 14 و 9 جلسه تحت مداخله با این روش قرار گرفتند و بلافاصله پس از درمان و سه ماه پس از آن نیز ارزیابی شدند. افزایش خرانه واژگان بیانی از طبقه اسامی و افعال و ترکیبات کلمات به عنوان هدف گفتاری آن‌ها در نظر گرفته شد. داده‌ها به روش دیداری و با استفاده از مقیاس IRD (Improvement rate difference‎) تجزیه و تحلیل گردید. یافته‌ها: هر 3 کودک در مهارت‌های زبانی خود در مرحله مداخله، جلسات حفظ و سه ماه پس از درمان، در مقایسه با ارزیابی‌های اولیه در خط پایه، پیشرفت قابل توجهی را نشان دادند. ضرایب تأثیر در رفتارهای بیان اسامی، بیان افعال و ترکیب‌های واژگانی به ترتیب برای کودک الف؛ 1، 91/0 و 77/0، برای کودک ب؛ 92/0، 77/0 و 60/0 و برای کودک ج؛ 1، 1 و 66/0 به دست آمد. نتیجه‎گیری: آموزش استراتژی‌های فراگیر پیشرفته به والدین کودکان مبتلا به تأخیر زبانی، می‌تواند تأثیر بسزایی در بهبود مهارت‌های زبان بیانی این کودکان داشته باشد.

Title: مروری بر تأثیر روش Dry needling در دردهای میوفاشیال

http://jrrs.mui.ac.ir/index.php/jrrs/article/view/1952

دردهای میوفاشیال یکی از شایع‌ترین دردهای عضلانی یا فاشیای مربوط به آن‌ها می‌باشد که اغلب با Myofascial trigger point (MTP) همراه است. روش‌های متعددی برای تسکین دردهای مزمن MTP به کار گرفته شده است، اما هیچ یک به تنهایی در درمان موفق نبوده‌اند. سوزن خشک (Dry needling) یکی دیگر از روش‌هایی به شمار می‌رود که در چند سال اخیر برای درمان MTPها مورد استفاده قرار گرفته است. این روش، یک روش با حداقل تهاجم و با استفاده از سوزن طب سوزنی در عضلات اسکلتی می‌باشد. پژوهش حاضر مروری بر تحقیقاتی بود که روش Dry needling را برای درمان MTP معرفی کرده و میزان تأثیر این روش در بهبود دردهای میوفاشیال را بررسی نموده‌ بودند. روش Dry needling روش درمانی به نسبت جدیدی است و برای روشن‌تر شدن مکانیزم‌های تأثیر و روش استفاده، چه از لحاظ تعداد نقاط در هر جلسه و یا تعداد جلسات درمانی و زمان ماندن سوزن در عضله، نیاز به تحقیقات بیشتری دارد.

Title: بررسی مروری تأثیر انواع بوتولینوم توکسین در کاهش اسپاستیسیتی اندام فوقانی و تحتانی و بهبود Gait در افراد دچار سکته مغزی

http://jrrs.mui.ac.ir/index.php/jrrs/article/view/2281

مقدمه: سکته مغزی آسیب عصبی حاد ناشی از اختلال یا عدم خون‌رسانی به قسمتی از بافت مغز و سیستم عصبی است که ناشی از انسداد عروق مغزی به وسیله یک لخته خون و یا پارگی یکی از عروق تغذیه کننده آن قسمت از بافت مغز می‌باشد. این ضایعه باعث ایجاد اختلالات عملکردی متفاوتی می‌گردد که اسپاستیسیتی از جمله این عوارض به شمار می‌رود. بوتولینوم توکسین به عنوان روش مؤثری در کاهش اسپاستیسیتی شناخته شده است. هدف از انجام این مطالعه، مروری بر مطالعات صورت گرفته در ارتباط با کاربردهای متفاوت بوتولینوم توکسین، تأثیرگذاری، نتایج و نوع کاربرد این روش درمانی بود. مواد و روش‌ها: با استفاده از کلمات کلیدی سکته مغزی، اسپاستیسیتی و بوتولینوم توکسین، مقالات مرتبط بدون محدودیت زمانی، از پایگاه‌های Scopus، Elsevier، Science Direct، Springer، MEDLINE و PubMed استخراج شدند. یافته‌ها: 193 مقاله از پایگاه‌های ذکر شده به دست آمد و با توجه به معیارهای ورود و خروج، 36 مقاله که به توصیف کاربرد بوتولینوم توکسین در کاهش اسپاستیسیتی و بهبود کارکرد حرکتی اندام‌ها و ارتقای پارامترهای Gait پرداخته بودند، مورد بررسی قرار گرفت. با توجه به مطالعات انجام شده، این تزریق باید به همراه درمان‌های دیگری از جمله کاردرمانی، فیزیوتراپی و تمرینات خانه‌محور به کار رود تا بهبود شایانی در توانمندی فرد مبتلا داشته باشد. نتیجه‌گیری: بوتولینوم توکسین یک درمان تأثیرگذار است که به همراه تکنیک‌های مورد استفاده در توان‌بخشی، جهت کاهش اسپاستیسیتی ناشی از اختلالات مغزی به کار می‌رود. بنابراین، بهبود در کارکردهای حرکتی کیفیت زندگی را به همراه دارد.
